# The mediating role of PERMA in the relationship between social role engagement and drug use problems among veterans with mental illness

**DOI:** 10.3389/fpsyt.2025.1571625

**Published:** 2025-09-17

**Authors:** Rebecca Campa, Guillermina R. Solis, Muharrem Koc, Teresa A. Granger, Beatrice Lee, Emre Umucu

**Affiliations:** ^1^ Department of Public Health Sciences, The University of Texas at El Paso, El Paso, TX, United States; ^2^ College of Nursing, The University of Texas at El Paso, El Paso, TX, United States; ^3^ Department of Special Education and Rehabilitation Psychology, The University of Wisconsin-Madison, Madison, WI, United States; ^4^ Department of Educational Studies in Psychology, Research Methodology, and Counseling, University of Alabama, Tuscaloosa, AL, United States; ^5^ Tuscaloosa VA Medical Center, Research & Development, Tuscaloosa, AL, United States; ^6^ Department of Rehabilitation Science, The University of Texas at El Paso, El Paso, TX, United States; ^7^ South Texas VA Medical Center, San Antonio, TX, San Antonio, TX, United States

**Keywords:** veterans, substance use disorders (SUDs), PERMA model, drug use, positive psychology

## Abstract

**Background:**

Substance use disorders (SUDs) are a critical issue among veterans, often co-occurring with mental illnesses and negatively impacting their well-being. This study explores the mediating role of well-being, as conceptualized by the PERMA model, in the relationship between ability to engage in social roles and activities and degree of problems related to drug abuse among veterans.

**Methods:**

Data were collected from 156 veterans with self-reported mental health conditions. A parallel multiple mediation analysis using PROCESS macro assessed the role of PERMA components in mediating the relationship between ability to engage in social roles and activities and degree of problems related to drug abuse.

**Results:**

Ability to engage in social roles and activities was positively correlated with overall well-being (r = .49, p <.05) and negatively correlated with degree of problems related to drug abuse (r = −.19, p <.05). Mediation analysis revealed that PERMA fully mediated the relationship between ability to engage in social roles and activities and degree of problems related to drug abuse, with accomplishment emerging as a significant indirect pathway (Effect = −0.05). The model explained 17% of the variance in degree of problems related to drug abuse.

**Conclusions:**

Findings underscore the importance of well-being, particularly accomplishment, in the relationship between veterans’ ability to engage in social roles and activities and degree of problems related to drug abuse. Interventions integrating well-being frameworks may offer holistic approaches to recovery for veterans with SUDs and mental illnesses. Future research should explore longitudinal effects of PERMA-focused interventions.

## Introduction

Mental illness represents a substantial public health concern, affecting millions of U.S. adults, including both civilian and veteran populations, each year (1–4). In 2021, 23.1% of U.S. adults—equivalent to 59.3 million individuals, or 1 in 5—experienced mental illness (5). Among veterans, the prevalence of mental health disorders is notably higher, with research attributing this disparity to unique challenges faced during military service, such as prolonged separation from loved ones, exposure to combat stressors, and trauma from witnessing harm (6, 7). Addressing veterans’ mental health concerns requires a comprehensive conceptualization of co-occurring conditions, particularly the documented link between substance use disorders (SUD) and mental illness, which disproportionately impacts this population (8, 9).

Over 1 in 10 veterans meet the diagnostic criteria for a SUD, a prevalence exceeding that of civilians (10). Upon their initial visit to Department of Veterans Affairs (VA) medical facilities, approximately 11% of veterans are diagnosed with SUD, with alcohol misuse affecting over 80%, illegal drug use affecting nearly 27%, and dual use of alcohol and illegal drugs affecting approximately 7% (11). Moreover, heroin addiction has emerged as a particularly pervasive issue, with 45,000 veterans diagnosed in 2018 alone (11, 12). It is also important to highlight veterans have been significantly affected by the opioid overdose crisis, experiencing a 53% rise in drug overdose death rates between 2010 and 2019 (13). Researchers also reported that the risk of opioid overdose among veterans should be viewed as the outcome of a continuous interplay between biological, psychological, social, and structural factors.

Prior research has illuminated key variables influencing the prevention and treatment of SUDs (14–16), with loneliness emerging as a particularly significant factor. Hosseinbor et al. (17) found that individuals with SUDs reported significantly higher levels of loneliness in familial and romantic relationships compared to those without addiction. Ashrafioun et al. (18) explored opioid use disorder and its relation to difficulties in participating in social roles and reported those with opioid use disorder were significantly more likely to report severe difficulties in social participation compared to those without opioid use disorder, and among those with opioid use disorder, such difficulties were linked to suicidal ideation, showing the importance of social engagement and community participation.

The social environment is a key factor in shaping an individual’s risk of drug use and the development of a drug use disorder (19). Veterans, especially those with mental illness, face substantial challenges reintegrating into civilian life and community participation (20). One of the critical determinants of successful community reintegration is the ability to engage in meaningful social roles which have been consistently linked to psychological well-being (1, 21). Conversely, when veterans face barriers to achieving these social roles, they may prefer maladaptive coping strategies, including substance use. For example, Alverson et al. (22) examined the longitudinal course of substance abuse and reported that regular community engagement in enjoyable activities were strongly linked to sustained abstinence in individuals with SUD.

Recently, positive psychology has emerged as a promising framework in the treatment of SUD. Stone (23) advocated for integrating positive psychology principles into SUD treatment, emphasizing a focus on enhancing well-being and quality of life rather than solely on abstinence. This approach may reduce stigma and increase engagement. Although positive psychology shows promise in improving outcomes for veterans with SUD, more research is needed to explore how specific elements of Seligman’s (24) PERMA model may help reducing substance use problems among veterans with mental illness. Therefore, the purpose of this study is to explore whether PERMA factors mediate the relationship between veterans’ ability to engage in meaningful social roles and activities and degree of problems related to drug abuse.

## Methods

### Procedures

This study received exempt status approval from the Institutional Review Board at the University of Texas at El Paso. Participants were recruited through Amazon Mechanical Turk (MTurk), a crowdsourcing platform that allows individuals with MTurk accounts to complete various Human Intelligence Tasks (HITs). MTurk has been recognized as a reliable, valid, diverse, cost-effective, and large-scale method for data collection (25–27). It has previously been utilized in rehabilitation research (e.g., 1, 26, 28–39). According to Lund and colleagues (26), MTurk may serve as an effective platform for recruiting American adults with disabilities (p. 7). Lund and colleagues (26) reported that “Amazon’s MTurk may be a good platform with which to recruit American adults with disabilities” (p. 7). The MTurk has options to restrict participants based on specific criteria (e.g., location). In this study, participation was restricted to individuals residing in the United States who had military experience. Participants completed an online survey hosted on Qualtrics, and only those who were 18 years or older and self-reported a mental health condition were included. Before participating, all individuals reviewed and agreed to an online consent form. Upon survey completion, participants were compensated with $4.00.

### Measures

#### Sociodemographic survey

In order to collect information about participants’ age, gender, educational background, the nature of their mental illness and prior military experience, we employed a sociodemographic survey.

#### Ability to participate in social roles and activities

The PROMIS^®^ Item Bank v2.0—Ability to Participate in Social Roles and Activities—Short Form 4a was used to assess participants’ ability to engage in social roles and activities (40). This measure consists of four items, where respondents rate their perceived ability to perform social functions on a scale from 1 (always) to 5 (never). Higher scores reflect greater perceived ability to participate. Raw scores were converted to standardized T-scores for interpretation.

#### PERMA profiler

Well-being in this study was assessed using the PERMA Profiler, a scale based on Seligman’s five pillars of well-being: positive emotion, engagement, relationships, meaning, and accomplishment (41). These five domains are each measured by three items. Participants responded on an 11-point Likert scale, ranging from 0 (never) to 10 (always), or 0 (not at all) to 10 (completely). Higher scores indicate greater well-being in each domain.

#### Drug Abuse Screening Test-10

DAST-10 is a brief, self-report tool used to assess degree of problems related to drug abuse (42). The test includes 10 yes/no questions designed to screen for drug use and its impact on the individual’s life. Each positive answer (yes) is assigned one point, with the total score ranging from 0 to 10. A score of 1–2 indicates “at risk” drug use, suggesting the individual would benefit from a brief intervention; a score of 3–5 reflects “moderate” drug abuse, requiring brief treatment, while a score of 6 or higher points to “severe” drug abuse, necessitating a referral for more intensive treatment.

### Data analysis

We conducted preliminary analyses of descriptive statistics and correlation coefficients using SPSS 30.0 and the PROCESS macro for SPSS (43). A parallel multiple mediation analysis was performed to assess the roles of PERMA as mediators in the relationship between the ability to engage in social roles and activities and problems related to drug abuse. Additionally, we employed the bootstrap testing method to examine the mediation hypothesis. Researchers (44, 45) reported that bootstrap resampling technique generate distributions of indirect effect estimates, which are then used to construct confidence intervals.

## Results

### Participants and descriptive statistics

The study included 156 veterans, with an average age of 37.9 years (SD = 10.64). A majority of participants were male (66.7%), and most identified as White (70%) and married (52.6%). Educationally, 39% held a bachelor’s degree, with others reporting varying levels of education, including some college credit without a degree (23.1%), an associate’s degree (13.5%), a graduate degree (12.2%), a trade/technical/vocational training (7.1%), and high school degree (5.8%). Participants reported experiencing at least one mental health condition, including depression (64.7%), anxiety (57.1%), PTSD (35.3%), substance use disorder (10.3%), bipolar disorder (4.5%), personality disorder (4.5%), and other mental health conditions (3.9%). Participants reported a moderate level of well-being (*M* = 5.91, *SD* = 2.13). Ability to participate in social roles and activities T-scores ranged from 31.80 to 64.20, with an average of *M* = 47.78 (*SD* = 8.40). DAST-10 scores ranged from 0 to 10, with an average of *M* = 1.50 (*SD* = 2.53).

### Correlation analysis

The ability to participate in social roles and activities was positively correlated with well-being (*r* = .49, *p* < 0.05) and negatively correlated with the degree of problems related to drug abuse (*r* = −.19, *p* < 0.05). Additionally, well-being was negatively correlated with the degree of problems related to drug abuse (*r* = −.34, *p* < 0.05).

### Mediation analysis

The total effect of ability to participate on the degree of problems related to drug abuse was significant (*B* = −.06, *p* <.05, 95% CI [−0.11, −0.01]), indicating a negative relationship. The direct effect was not significant (*B* = −0.001, *p* = .7029, 95% CI [−0.0613, 0.0414]), suggesting full mediation by the PERMA components. The total indirect effect of the PERMA components was significant (Effect = −0.05, 95% CI [−0.09, −0.01]), with accomplishment emerging as the strongest contributor to the mediation (*β*= −.40, *p*<0.05). These findings underscore the importance of accomplishment as a key factor linking social engagement to reduced problems related to drug abuse. Overall, the model explained 17% of the variance in the degree of problems related to drug abuse (*R*² = .17).

## Discussion

This study identified several significant relationships, including a positive relationship between veterans’ ability to engage in social roles and activities and their well-being as measured by the PERMA model, a negative relationship between PERMA and problems related to problems related to drug abuse, and a negative relationship between the ability to engage in social roles and activities and problems related to problems related to drug abuse. These results align with Seligman’s (24) PERMA framework, which highlights the interconnection of PERMA as essential components of well-being. For example, positive emotions may provide alternative sources of satisfaction with life, reducing reliance on substances for temporary relief and coping (46). Healthy engagement is often replaced by substance use when mental health challenges hinder opportunities for genuine social engagement (47, 48). Community participation, volunteer experiences, and family roles may serve as a protective factor against drug use by fostering a sense of accomplishment (1, 49–53).

Our study also revealed a significant negative relationship between veterans’ ability to engage in social roles and activities and problems related to drug abuse, aligning with prior research that highlights the protective effects of social connectedness (19, 54). Veterans with mental illness often experience isolation during the transition to civilian life (1, 20, 55, 56), increasing vulnerability to substance use as a maladaptive coping mechanism (57). Active community participation may foster healthier coping strategies, mitigating the adverse effects of social isolation that is associated with increased substance use (19, 54).

Finally, PERMA was found to mediate the relationship between problems related to drug abuse and the ability to engage in social roles and activities. Specifically, only the accomplishment component of PERMA significantly correlated with the lower problems related to drug abuse when accounting for all mediators and the direct effect of ability to participate in social roles and activities. This indicates that a sense of accomplishment and goal attainment can serve as protective factors against drug use problems among veterans with mental illness. It is important to highlight that while some individuals might experience a temporary sense of meaning or accomplishment through substance use, such as feelings of social acceptance, these effects ultimately undermine opportunities for healthy engagement in life activities (58, 59). In contrast, genuine accomplishments that align with one’s personal values and goals are linked to improved well-being (24).

Improving a sense of accomplishment, as indicated as a malleable protective factor (24), may be a particularly effective strategy for reducing problems related to drug abuse since community participation and engagement interventions are available for veterans with mental illness. Increasing social engagement and community participation alone may not be enough to reduce drug abuse and associated problems; hence, clinicians should help veterans define and attain goals to have stronger protective effects of social engagement. Interventions and strategies that improve goal setting and mastery experiences may thus serve as valuable strategy of substance use prevention or recovery for veterans with mental illness.

Although there are many clinical implications, one important clinical implication we want to highlight is the potential value of meaningful employment in supporting veterans’ recovery from drug use and associated negative consequences. Employment may support veterans with mental illness in developing productive social roles that align with their life values and goals, which may eventually improve their sense of accomplishment (1, 21, 60–67). Importantly, employment can serve as a powerful vehicle for fostering a sense of purpose and social contribution (1, 60)—all of which are central to the PERMA framework and have been shown to protect against drug use. It is also important to recognize that not all employment is equally beneficial. Simply having a job may not lead to improved well-being if the work is stressful and misaligned with the veteran’s interests and strengths. Therefore, strengths-based positive psychology techniques may help veterans obtain meaningful employment by aligning job roles with their interests and strengths, thereby supporting community reintegration and smoother transition, enhancing a sense of achievement, and ultimately reducing the risk of drug abuse (68, 69).

Despite the strengths of our work, several limitations warrant consideration. The reliance on self-reported measures may cause response biases, including social desirability. Besides, the cross-sectional design limits the ability to draw causal inferences regarding relationships among social role engagement, well-being, and drug use. Future research should employ longitudinal to better conceptualize causal pathways and the long-term impact of enhancing well-being on substance use reduction. Finally, the PERMA focuses primarily on individual-level psychological factors, limiting our understanding of the social and structural determinants of drug use among veterans with mental illness. Models and theories integrating the social determinants of health model may provide a more holistic approach understanding the relationship between ability to participate in social roles and activities and problems related to drug abuse.

## Data Availability

Due to IRB requirements, we do not have data shared online. Requests to access the datasets should be directed to EU at eumucu@utep.edu.
